# Appendiceal Endometriosis: A Rare Case of Endometriosis Mimicking Acute Appendicitis

**DOI:** 10.7759/cureus.81280

**Published:** 2025-03-27

**Authors:** Riah S Lee, Yasmine Hemida, Douglas James

**Affiliations:** 1 Surgery, Touro College of Osteopathic Medicine, Middletown, USA; 2 General Surgery, Garnet Health Medical Center, Middletown, USA; 3 Trauma and Acute Care Surgery, Garnet Health Medical Center, Middletown, USA

**Keywords:** appendiceal endometriosis, appendix endometriosis, diagnosing appendicitis, diagnosis of acute appendicitis, extrauterine endometriosis, intrauterine devices (iud), negative appendectomy

## Abstract

Appendiceal endometriosis (AE) is a rare type of extragonadal endometriosis with symptoms of right lower abdominal pain, nausea, and vomiting that mimic acute appendicitis. The gold standard for a definitive diagnosis is a histopathological examination of the excised appendix. We report a case of AE in a 39-year-old female patient, G10P3, with a past surgical history of cholecystectomy, seven dilation and curettage procedures, and one prior cesarean section presenting with a right lower quadrant pain with intermittent non-bloody diarrhea, nausea, and vomiting that is not exacerbated by movement. The patient was mildly tachycardic with otherwise stable vitals and no leukocytosis. The beta-hCG test was negative with a CT-confirmed Mirena® intrauterine contraceptive device (IUD) (Bayer AG, Leverkusen, Germany) placement. The patient denied heavy bleeding or vaginal discharge. The CT scan of the abdomen and pelvis with oral contrast demonstrated findings suggestive of appendicitis, leading to a subsequent laparoscopic appendectomy. The resected specimen showed histopathology features of endometriosis, confirming AE. AE poses diagnostic challenges due to its nonspecific imaging findings along with variable symptomatic presentations. The recommended management of AE is an appendectomy with a gynecological follow-up postoperatively. AE is a rare condition that can masquerade as acute appendicitis in female patients. We highlight the importance of including AE in the differential diagnosis of female patients presenting with lower abdominal pain.

## Introduction

Acute abdominal pain is one of the leading complaints among emergency department (ED) visits in the United States, accounting up to 8.8% of all ED visits [1]. In the majority of cases with patients presenting with right lower abdominal pain, appendicitis is contemplated as the initial differential diagnosis. With clinical findings and computed tomography of the abdomen and pelvis to guide the diagnosis of appendicitis, appendectomy remains the most common emergency abdominal surgical procedure [2]. 

While appendectomy is frequently performed, a recent study reports a negative appendectomy rate between 15-39% [3]. Negative appendectomy is often associated with longer hospitalization, higher morbidity, and higher cost [4]. Therefore, it warrants a deep understanding of the importance of the reduction in the negative appendectomy rate and establishing a correct preoperative diagnosis. Other common causes of right lower abdominal pain include inflammatory bowel disease, cecal diverticulitis, ruptured ectopic pregnancy, pelvic inflammatory disease, and, rarely, appendiceal endometriosis (AE). 

Endometriosis is characterized by the presence of ectopic endometrial tissue outside of the uterine cavity, usually in the ovaries and pelvic peritoneum. In rare cases, these endometrial nodules can be found in the appendix, mimicking symptoms of acute appendicitis. AE is extremely uncommon, and its preoperative diagnosis is challenging. Herein, we illustrate a case of AE that was preoperatively misdiagnosed as acute appendicitis.

## Case presentation

A 39-year-old female patient, G10P3, with a past surgical history of cholecystectomy, seven dilation and curettage procedures, and one prior cesarian section presented with an acute right lower quadrant pain, intermittent diarrhea, nausea, and vomiting that was not exacerbated by movement. Although the symptoms began approximately 12 hours prior, the patient reported having similar symptoms of nausea and diarrhea two weeks ago that subsided with rest. 

**Figure 1:**
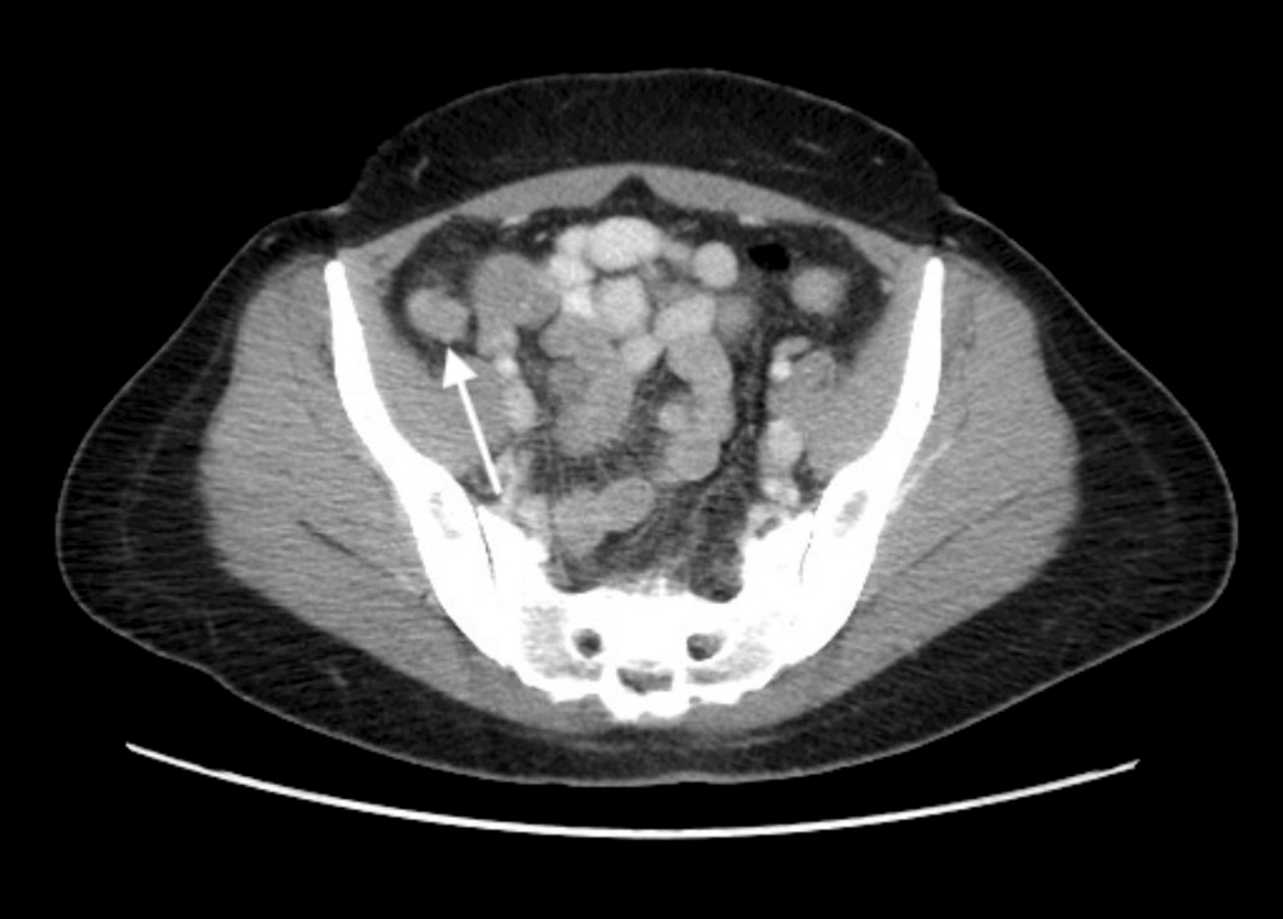
Cross-sectional view of CT of the abdomen and pelvis showing mild thickening and inflammatory changes of the appendix without evidence of perforation.

**Figure 2:**
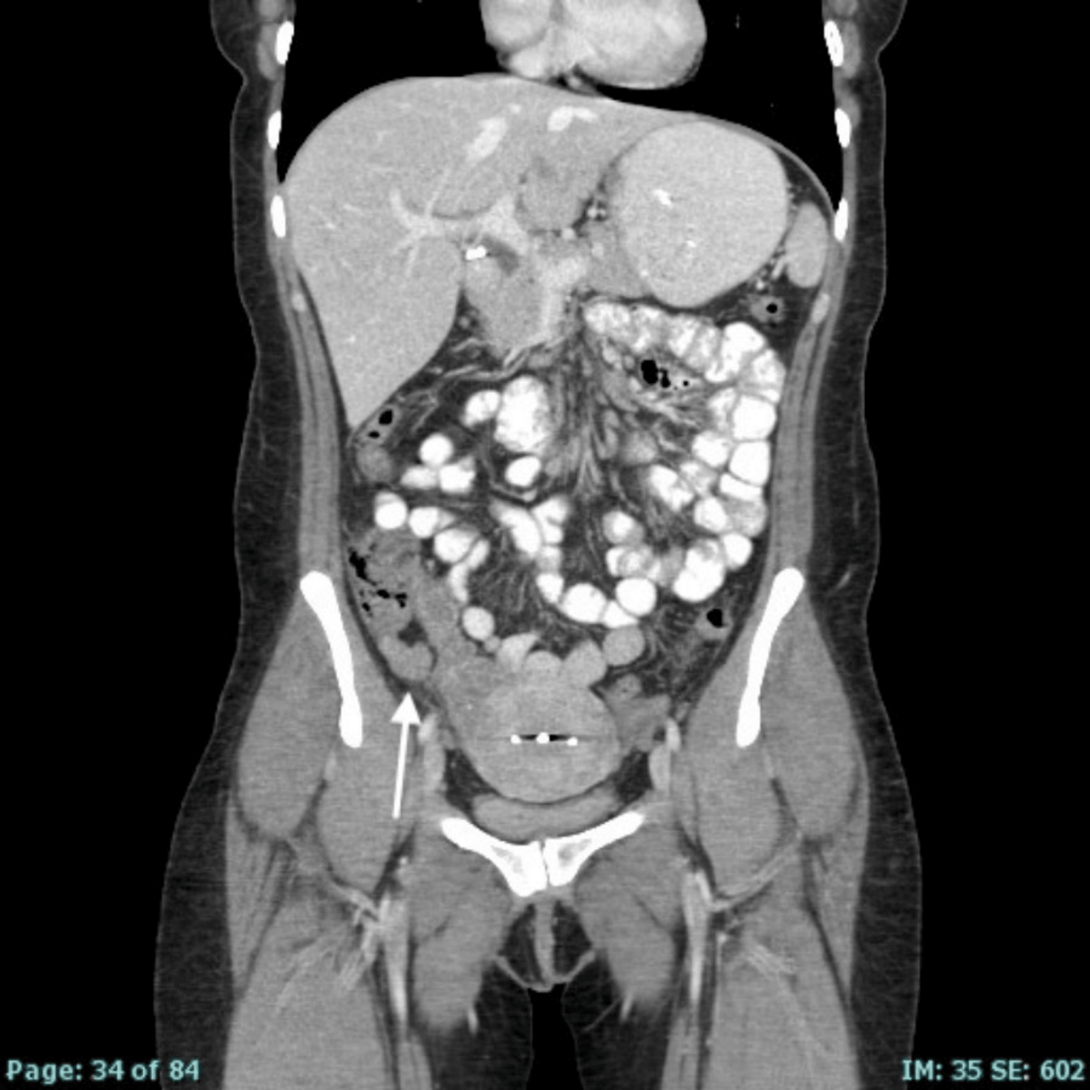
Coronal view of CT of the abdomen and pelvis suggesting thickening of the appendix.

The patient had a heart rate of 110 beats per minute but otherwise stable vitals with a white blood cell count of 9,800/mm^3^. Urinalysis was tested normal and the last menstrual cycle was reported to be three months ago. The beta-human chorionic gonadotropin (hCG) test was negative with a CT-confirmed levonorgestrel-releasing intrauterine system (Mirena® intrauterine contraceptive device (IUD); Bayer AG, Leverkusen, Germany) placement. The patient denied heavy bleeding or vaginal discharge. Upon physical examination, the abdomen was soft, non-distended, and tender to palpation with pain traveling from the right lower quadrant to the right pelvis. There were negative obturator and Rovsing's signs but a positive Mcburney's sign. Additionally, there was no guarding, rebound tenderness, or rigidity. The calculated Alvarado score was 4. While ultrasound images showed no acute findings, a CT scan of the abdomen and pelvis with oral contrast demonstrated mild thickening and inflammation of the appendix, as seen in Figures 1, 2. Hence, laparoscopic appendectomy was performed. Intraoperatively, the tip of the appendix showed thickening with inflammation and omental adhesions. There were no ascites, peritoneal deposits, or any signs of perforation. Ectopic endometrial implantation or other pathologies were not observed throughout the abdomen.

**Figure 3:**
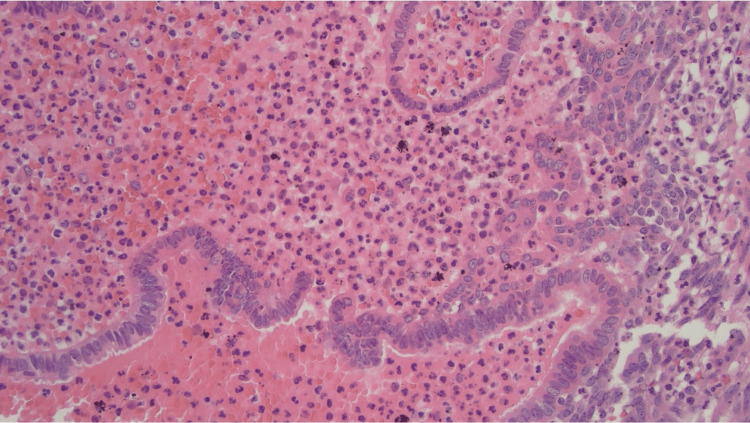
Acute inflammation associated with endometriosis (200x).

**Figure 4:**
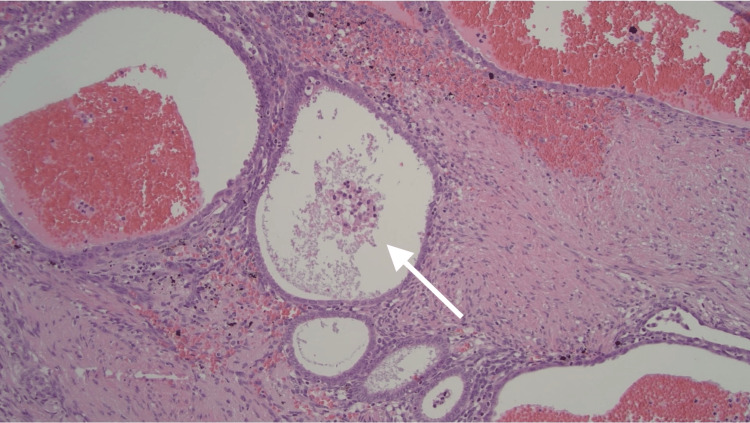
Endometriosis with acute and chronic hemorrhage as evidenced by hemosiderin laden macrophages (100x).

**Figure 5:**
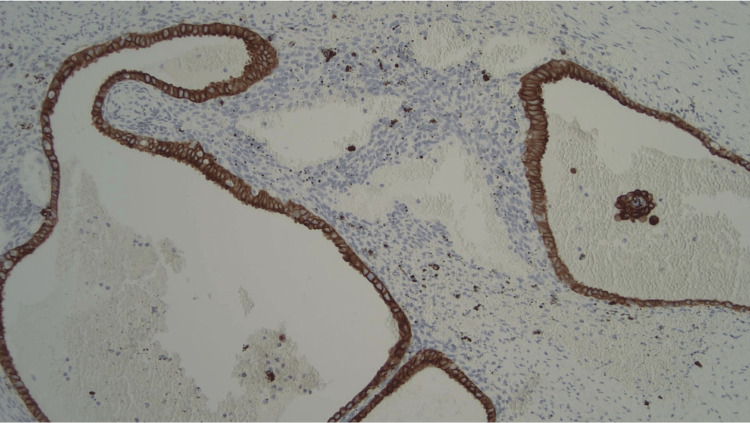
Endometrial-type glands staining positive for cytokeratin 7 (CK7).

**Figure 6:**
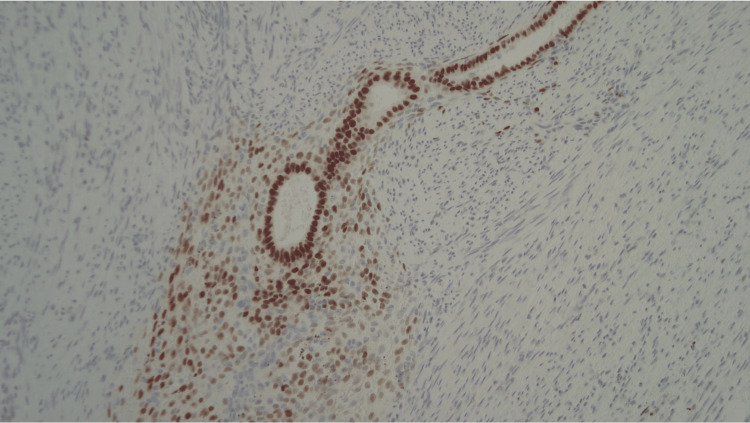
Endometrial glands and stroma positive for estrogen receptor (ER) stain.

**Figure 7:**
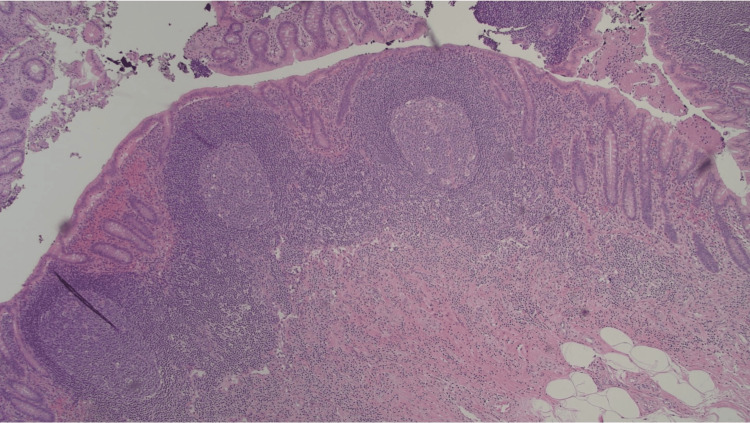
Appendiceal lumen without evidence of acute appendicitis (40x).

The pathological examination revealed a focal, nonspecific acute inflammation on the endometrial nodule, along with serosal adhesions (Figure 3). The specimen showed features of both acute and chronic hemorrhage, as evidenced by hemosiderin-laden macrophages (Figure 4). Immunohistological stains for the glands were focally positive for cytokeratin (CK) 7 and estrogen receptor (ER) and negative for CK20 and CDX2, which were consistent with endometriosis (Figures 5, 6). The appendix itself showed no acute appendicitis (Figure 7). No dysplasia or malignancy was noted. Postoperative recovery was uneventful, and the patient was subsequently discharged on the same day with no residual pain. The patient was referred to a gynecologist for further assessment.

## Discussion

Endometriosis is one of the most perplexing gynecological conditions that influences 10-15% of all reproductive-age women [5]. It is characterized by the development of estrogen-dependent endometrial tissue outside the uterine cavity, the most common being in the ovaries, fallopian tubes, and pelvic peritoneum [6]. While the clinical presentation of endometriosis varies in women, common symptoms include persistent pelvic pain, infertility, dysmenorrhea, dyspareunia, dyschezia, and dysuria [7].

AE is extremely rare, with a prevalence ranging from 0.05% to 1.7% [8,9]. The symptoms of AE are highly variable and may mimic acute appendicitis with symptoms of nausea, vomiting, right lower quadrant abdominal colic, and melena [10]. Currently, there are no specific diagnostic procedures available for the preoperative diagnosis of AE. While the patient’s medical history, physical examination, cancer antigen 125 (CA-125) marker, colonoscopy, transvaginal, transrectal ultrasonography, barium enema, CT, and MRI can be utilized to diagnose endometriosis, they provide limited value for the diagnosis of AE. The gold standard for the diagnosis of AE is laparoscopy with a confirmatory histopathologic examination [11,12].

Approximately half of the AE involves the tip of the appendix, while the other half involves the body. It is reported that muscular and seromuscular involvement occurs in two-thirds of patients, whereas the remaining one-third of the patients have a serosal-layer involvement [13]. The patient in the current case falls into the latter category, with the involvement of the serosal layer. The histopathological features of endometriosis include the presence of endometrial glands, stroma, fibrosis, hemosiderin-laden macrophages, and signs of inflammation [14]. While these features are suggestive of endometriosis, immunohistochemical stains with a panel of CK7, ER, CK20, and CDX2 antibodies can be utilized to distinguish difficult cases of endometriosis [15]. As for the current case, endometrial glands were stained positive for CK7 and ER and negative for CK20 and CDX2. These findings were consistent with endometriosis.

Depending on the severity and type of symptoms, treatment options vary for patients with endometriosis. It is estimated that 20-25% of women of reproductive age with endometriosis are asymptomatic [16]. In contrast, our patient exhibited symptoms resembling acute appendicitis, such as intermittent right lower abdominal pain accompanied by non-bloody diarrhea, nausea, and vomiting. Currently, there is limited consensus on the practice guidelines for appendiceal endometriosis. However, surgical excision of the involved tissue of endometriosis remains the most efficient treatment for endometriosis compared to hormonal therapy [10,17].

Previously, AE was believed to be associated with ovarian endometriosis [18]. However, recent studies revealed multiple cases of AE without the involvement of ovarian endometriosis, highlighting a lack of association between AE and ovarian disease [19]. While surgical resection of AE reduces the overall pain, it warrants a close gynecological workup [20].

## Conclusions

AE is a rare entity that often mimics symptoms of acute appendicitis. Preoperative diagnosis is extremely challenging despite multiple imaging modalities. Regardless of the underlying etiology, appendectomy remains the standard of care due to the lack of a reliable preoperative diagnostic tool to discern AE. Currently, the definitive diagnosis is only established by the histopathologic examination of the appendix. Nevertheless, AE should be carefully considered in the differential diagnosis of patients with symptoms resembling acute appendicitis, especially in female patients with a history of previous gynecological procedures. 
